# Citation analysis: a new approach to assess the uptake of core outcome sets

**DOI:** 10.1186/1745-6215-16-S2-P50

**Published:** 2015-11-16

**Authors:** Karen Barnes, Mike Clarke, Jamie Kirkham, Paula Williamson

**Affiliations:** 1 University of Liverpool, Liverpool, UK; 2 Queen's University Belfast, Belfast, UK

## Background

A recent study [1] assessed uptake of a rheumatoid arthritis (RA) Core Outcome Set (COS), published in 1994 [2], and found that by 2010 over 70% of RA trials reported the COS. The study examined trial reports but this method has proved to be lengthy and does not include current information. The aim of this study is to develop an efficient approach to assessing COS uptake with results based on up to date information.

## Method

Citation analysis will be used to assess COS uptake. Citation reports for 198 published COS will be run in SCOPUS. CENTRAL (The Cochrane Central Register of Controlled Trials) will be used to identify those citations that are RCTs. Consideration will be given to how many of these RCTs reported all core outcomes.

## Results

Using RA as a motivating example, 202 citations were found using the RA COS published in 1994^2^. Fourteen citations were identified by CENTRAL as being RCTs. Full results for all COS will be presented.

## Conclusion

It is important to develop a method to measure uptake of COS efficiently to allow the impact of COS development research to be assessed. Citation analysis has the potential to offer an effective method to do this.

## References

[B1] Kirkham Outcome measures in rheumatoid arthritis randomised trials over the last 50 years Trials 2013 14 324 24103529 10.1186/1745-6215-14-324 PMC3852710

[B2] Boers M World Health Organization and International League of Associations for Rheumatology core endpoints for symptom modifying antirheumatic drugs in rheumatoid arthritis clinical trials J Rheumatol 1994 21 suppl 41 86 89 7799394

